# Supplementary material for: Breathwork and holistic wellbeing: A protocol for a scoping review
Source: PLoS One. 2025 Sep 30;20(9):e0333360. doi: 10.1371/journal.pone.0333360 (PMC12483246; doi:10.1371/journal.pone.0333360)
Supplement: S1 Appendix — (DOCX) [file pone.0333360.s001.docx]

**S1 Appendix. Search strategy**

A primary search string will be produced using the breathing interventions search terms, connected with the ‘OR’ operator. Secondary search strings will be produced using the search terms related to each wellbeing domain – individual, collective and planetary – using the ‘OR’ operator. A tertiary search string will be produced using search terms related to health conditions using the ‘OR’ operator. A search query will then be created which incorporates the primary search string and adjoining it to the secondary search string using the ‘AND’ operator. The tertiary search query will then be added using the ‘NOT’ operator. Refer to S2 Table for full database-specific search strategies.
