# Supplementary material for: Breathwork and holistic wellbeing: A protocol for a scoping review
Source: PLoS One. 2025 Sep 30;20(9):e0333360. doi: 10.1371/journal.pone.0333360 (PMC12483246; doi:10.1371/journal.pone.0333360)
Supplement: S2 Table — (DOCX) [file pone.0333360.s002.docx]

**S2 Table. Database-specific search strategies**

| **Database** | **Concept** | **Search** | **Query** |
| --- | --- | --- | --- |
| PubMed | Individual Wellbeing | #1 | ((((Wellbeing[Title] OR "well being"[Title] OR well-being[Title] OR flourish*[Title] OR self-connect*[Title] OR "connect* to self"[Title] OR "connect* with self"[Title] OR self-awareness[Title] OR self-accept*[Title] OR self-alignment[Title] OR "align* with self"[Title] OR self-determination[Title] OR self-efficacy[Title] OR self-esteem[Title] OR self-identity[Title] OR self-concept[Title] OR consciousness[Title] OR interoception[Title] OR "internal sensation*"[Title] OR "internal state*"[Title] OR intuition*[Title] OR "true self"[Title] OR "psychological* health"[Title] OR "emotional health"[Title] OR "mental health"[Title] OR "life satisfaction"[Title] OR "satisf* with life"[Title] OR "positive affect"[Title] OR "negative affect"[Title] OR "physical health"[Title] OR "positive emotion*"[Title] OR "negative emotion*"[Title] OR joy[Title] OR "heart rate variability"[Title] OR HRV[Title] OR hedoni*[Title] OR euda*moni*[Title] OR eudemon*[Title] OR happiness[Title] OR engag*[Title] OR meaning[Title] OR accomplishment[Title] OR achievement[Title] OR purpose[Title] OR "environmental mastery"[Title] OR "personal growth"[Title] OR matter*[Title] OR value*[Title] OR thriv*[Title] OR vitality[Title] OR resilience[Title] OR "quality of life"[Title] OR QoL[Title] OR autonomy[Title] OR competence[Title]) OR ("Psychological Well-Being"[MeSH Terms])) OR ("Quality of Life"[MeSH Terms])) OR ("heart rate"[MeSH Terms])) OR ("mental health"[MeSH Terms]) |
|  | Collective Wellbeing | #2 | (((((((((("social wellbeing"[Title] OR "social well-being"[Title] OR "social well being"[Title] OR "social capital"[Title] OR "social capacity"[Title] OR "social participation"[Title] OR "social functioning"[Title] OR "social actual*"[Title] OR "social inclusion"[Title] OR "social intel*"[Title] OR "social accept*"[Title] OR "social identi*"[Title] OR "social connect*"[Title] OR collective*[Title] OR psychosocial[Title] OR psycho-social[Title] OR collaboration[Title] OR affiliation*[Title] OR community[Title] OR cohesi*[Title] OR belong*[Title] OR benevolen*[Title] OR relat*[Title] OR "social support*"[Title] OR friendship*[Title] OR "connect* to others"[Title] OR prosocial[Title]) OR ("social status"[MeSH Terms])) OR ("social capital"[MeSH Terms])) OR ("social cohesion"[MeSH Terms])) OR ("social inclusion"[MeSH Terms])) OR ("social identification"[MeSH Terms])) OR ("Emotional Intelligence"[MeSH Terms])) OR ("social interaction"[MeSH Terms])) OR ("social participation"[MeSH Terms])) OR ("Interpersonal Relations"[MeSH Terms])) OR (Altruism[MeSH Terms]) |
|  | Planetary Wellbeing | #3 | "planetary wellbeing"[Title] OR "planetary well being"[Title] OR "planetary well-being"[Title] OR "environmental wellbeing"[Title] OR "environmental well being"[Title] OR "environmental well-being"[Title] OR "environment* attitude*"[Title] OR "environment* concern*"[Title] OR "environment* belief*"[Title] OR "environment* behav*"[Title] OR "environment* friend*"[Title] OR "environment* protect*"[Title] OR "environment* preserv*"[Title] OR "environment* steward*"[Title] OR "environment* identity"[Title] OR "environment* interdepend*"[Title] OR "ecological wellbeing"[Title] OR "ecological well-being"[Title] OR "ecological well being"[Title] OR "ecolog* attitude"[Title] OR "eco attitude"[Title] OR "ecolog* behave*"[Title] OR "eco behav*"[Title] OR "ecolog* concern"[Title] OR "eco concern"[Title] OR "ecolog* belief"[Title] OR "eco belief"[Title] OR pro-environment*[Title] OR "pro environment*"[Title] OR "nature preserv*"[Title] OR "nature connect*"[Title] OR "nature relat*"[Title] OR "nature protect*"[Title] OR "nature in self"[Title] OR "nature behav*"[Title] OR "preservation behave*"[Title] OR "protection behav*"[Title] OR sustainab*[Title] OR biophil*[Title] OR "bio phil*"[Title] OR "climate friend*"[Title] OR "climate conservation"[Title] OR "climate * attitude*"[Title] OR "climate * behav*"[Title] OR "climate * belief*"[Title] OR "climate * aware*"[Title] OR "conservation* behav*"[Title] |
|  | Holistic wellbeing | #4 | #1 OR #2 OR #3  ((((((Wellbeing[Title] OR "well being"[Title] OR well-being[Title] OR flourish*[Title] OR self-connect*[Title] OR "connect* to self"[Title] OR "connect* with self"[Title] OR self-awareness[Title] OR self-accept*[Title] OR self-alignment[Title] OR "align* with self"[Title] OR self-determination[Title] OR self-efficacy[Title] OR self-esteem[Title] OR self-identity[Title] OR self-concept[Title] OR consciousness[Title] OR interoception[Title] OR "internal sensation*"[Title] OR "internal state*"[Title] OR intuition*[Title] OR "true self"[Title] OR "psychological* health"[Title] OR "emotional health"[Title] OR "mental health"[Title] OR "life satisfaction"[Title] OR "satisf* with life"[Title] OR "positive affect"[Title] OR "negative affect"[Title] OR "physical health"[Title] OR "positive emotion*"[Title] OR "negative emotion*"[Title] OR joy[Title] OR "heart rate variability"[Title] OR HRV[Title] OR hedoni*[Title] OR euda*moni*[Title] OR eudemon*[Title] OR happiness[Title] OR engag*[Title] OR meaning[Title] OR accomplishment[Title] OR achievement[Title] OR purpose[Title] OR "environmental mastery"[Title] OR "personal growth"[Title] OR matter*[Title] OR value*[Title] OR thriv*[Title] OR vitality[Title] OR resilience[Title] OR "quality of life"[Title] OR QoL[Title] OR autonomy[Title] OR competence[Title]) OR ("Psychological Well-Being"[MeSH Terms])) OR ("Quality of Life"[MeSH Terms])) OR ("heart rate"[MeSH Terms])) OR ("mental health"[MeSH Terms]) OR ((((((((((("social wellbeing"[Title] OR "social well-being"[Title] OR "social well being"[Title] OR "social capital"[Title] OR "social capacity"[Title] OR "social participation"[Title] OR "social functioning"[Title] OR "social actual*"[Title] OR "social inclusion"[Title] OR "social intel*"[Title] OR "social accept*"[Title] OR "social identi*"[Title] OR "social connect*"[Title] OR collective*[Title] OR psychosocial[Title] OR psycho-social[Title] OR collaboration[Title] OR affiliation*[Title] OR community[Title] OR cohesi*[Title] OR belong*[Title] OR benevolen*[Title] OR relat*[Title] OR "social support*"[Title] OR friendship*[Title] OR "connect* to others"[Title] OR prosocial[Title]) OR ("social status"[MeSH Terms])) OR ("social capital"[MeSH Terms])) OR ("social cohesion"[MeSH Terms])) OR ("social inclusion"[MeSH Terms])) OR ("social identification"[MeSH Terms])) OR ("Emotional Intelligence"[MeSH Terms])) OR ("social interaction"[MeSH Terms])) OR ("social participation"[MeSH Terms])) OR ("Interpersonal Relations"[MeSH Terms])) OR (Altruism[MeSH Terms]) OR ("planetary wellbeing"[Title] OR "planetary well being"[Title] OR "planetary well-being"[Title] OR "environmental wellbeing"[Title] OR "environmental well being"[Title] OR "environmental well-being"[Title] OR "environment* attitude*"[Title] OR "environment* concern*"[Title] OR "environment* belief*"[Title] OR "environment* behav*"[Title] OR "environment* friend*"[Title] OR "environment* protect*"[Title] OR "environment* preserv*"[Title] OR "environment* steward*"[Title] OR "environment* identity"[Title] OR "environment* interdepend*"[Title] OR "ecological wellbeing"[Title] OR "ecological well-being"[Title] OR "ecological well being"[Title] OR "ecolog* attitude"[Title] OR "eco attitude"[Title] OR "ecolog* behave*"[Title] OR "eco behav*"[Title] OR "ecolog* concern"[Title] OR "eco concern"[Title] OR "ecolog* belief"[Title] OR "eco belief"[Title] OR pro-environment*[Title] OR "pro environment*"[Title] OR "nature preserv*"[Title] OR "nature connect*"[Title] OR "nature relat*"[Title] OR "nature protect*"[Title] OR "nature in self"[Title] OR "nature behav*"[Title] OR "preservation behave*"[Title] OR "protection behav*"[Title] OR sustainab*[Title] OR biophil*[Title] OR "bio phil*"[Title] OR "climate friend*"[Title] OR "climate conservation"[Title] OR "climate * attitude*"[Title] OR "climate * behav*"[Title] OR "climate * belief*"[Title] OR "climate * aware*"[Title] OR "conservation* behav*"[Title] |
|  | Breathing interventions | #5 | ("breathing intervention*"[Title] OR "breathing practice*"[Title] OR "breath* training"[Title] OR "breathing technique*"[Title] OR "breathing exercise*"[Title] OR "respiration practice"[Title] OR "breathing pattern"[Title] OR breathwork[Title] OR "breath* work"[Title] OR "control* respiration"[Title] OR "control* breath*"[Title] OR "diaphragm* breath*"[Title] OR "abdom* breath*"[Title] OR "thoracic breath*"[Title] OR "chest breath*"[Title] OR "clavicul* breath*"[Title] OR "rhythmic breath*"[Title] OR "functional breath*"[Title] OR "belly breath*"[Title] OR "deep breath*"[Title] OR "slow breath*"[Title] OR "fast breath*"[Title] OR "shallow breath*"[Title] OR "nostril breath*"[Title] OR "nose breath*"[Title] OR "mouth breath*"[Title] OR "pursed lip breath*"[Title] OR "pursed-lip breath*"[Title] OR "4 7 8 breath*"[Title] OR "4-7-8 breath*"[Title] OR "4 4 8 breath*"[Title] OR "4-4-8 breath*"[Title] OR "2-4 breath*"[Title] OR "five-finger breath*"[Title] OR "resonant breath*"[Title] OR "coherent breath*"[Title] OR "paced breath*"[Title] OR pranayama[Title] OR "yogic breath*"[Title] OR "yoga breath*"[Title] OR "sudarshan kriya yoga"[Title] OR kapalabhati[Title] OR "kapal bhati"[Title] OR "skull shining breath*"[Title] OR "nadi shodan*"[Title] OR "nadi shodhan*"[Title] OR "nadi sodhana"[Title] OR "alternate nostril breath*"[Title] OR "alternate nostril technique"[Title] OR "nadi sudi"[Title] OR "nadi suddhi"[Title] OR "nadi shuddhi"[Title] OR "anulom viloma"[Title] OR viloma[Title] OR "anulomviloma"[Title] OR "nerve purify* breath*"[Title] OR "channel cleaning breath*"[Title] OR ujjayi[Title] OR "ocean breath*"[Title] OR "victorious breath*"[Title] OR bhramari[Title] OR "bee breath*"[Title] OR "humming breath*"[Title] OR dirga[Title] OR dirgha[Title] OR "three part breath*"[Title] OR "three-part breath*"[Title] OR "circular breath*"[Title] OR "infant breath*"[Title] OR bhastrika[Title] OR "bellow* breath*"[Title] OR sitali[Title] OR sithali[Title] OR sheetali[Title] OR "cooling breath*"[Title] OR sheetkari[Title] OR sitkari[Title] OR "sipping breath*"[Title] OR "hissing breath*"[Title] OR samavritti[Title] OR "sama vritti"[Title] OR "square breath*"[Title] OR "equal breath*"[Title] OR "box breath*"[Title] OR plavini[Title] OR "floating breath*"[Title] OR "murcha breath*"[Title] OR "moorcha breath*"[Title] OR "swooning breath*"[Title] OR "surya bhedana"[Title] OR suryabhedana[Title] OR "sun piercing breath*"[Title] OR "surya nadi"[Title] OR "chandra bhedana"[Title] OR chandrabhedana[Title] OR "moon piercing breath*"[Title] OR "chandra nadi"[Title] OR "sukka purvaka"[Title] OR "shwasa prashwasa"[Title] OR "gita prana*"[Title] OR "sukha prana*"[Title] OR "easy* breath*"[Title] OR "nisshesha rechaka kumbhaka"[Title] OR shunyaka[Title] OR "bahya"[Title] OR "complete exhal*"[Title] OR "outer breath retention"[Title] OR "antara kumbhaka"[Title] OR "internal breath retention"[Title] OR "kevala kumbhaka"[Title] OR "absolute retention"[Title] OR "spontaneous retention"[Title] OR "complete retention"[Title] OR "breath of fire"[Title] OR "agni prana"[Title] OR "fire breath*"[Title] OR "simha* breath*"[Title] OR "lion* breath*"[Title] OR "qigong breath*"[Title] OR "wave breath*"[Title] OR "xingqi"[Title] OR "circulating breath*"[Title] OR "kidney breathing"[Title] OR "lower back breath*"[Title] OR "conscious* connected breath*"[Title] OR "quantum light breath*"[Title] OR "tummo"[Title] OR "pico pico breath*"[Title] OR "piko piko breath*"[Title] OR "soma breath*"[Title] OR "wim hof method"[Title] OR "oxygen advantage"[Title]) OR ("breathing exercises"[MeSH Terms]) |
|  | Holistic wellbeing AND breathing interventions | #6 | #4 AND #5  (((((((Wellbeing[Title] OR "well being"[Title] OR well-being[Title] OR flourish*[Title] OR self-connect*[Title] OR "connect* to self"[Title] OR "connect* with self"[Title] OR self-awareness[Title] OR self-accept*[Title] OR self-alignment[Title] OR "align* with self"[Title] OR self-determination[Title] OR self-efficacy[Title] OR self-esteem[Title] OR self-identity[Title] OR self-concept[Title] OR consciousness[Title] OR interoception[Title] OR "internal sensation*"[Title] OR "internal state*"[Title] OR intuition*[Title] OR "true self"[Title] OR "psychological* health"[Title] OR "emotional health"[Title] OR "mental health"[Title] OR "life satisfaction"[Title] OR "satisf* with life"[Title] OR "positive affect"[Title] OR "negative affect"[Title] OR "physical health"[Title] OR "positive emotion*"[Title] OR "negative emotion*"[Title] OR joy[Title] OR "heart rate variability"[Title] OR HRV[Title] OR hedoni*[Title] OR euda*moni*[Title] OR eudemon*[Title] OR happiness[Title] OR engag*[Title] OR meaning[Title] OR accomplishment[Title] OR achievement[Title] OR purpose[Title] OR "environmental mastery"[Title] OR "personal growth"[Title] OR matter*[Title] OR value*[Title] OR thriv*[Title] OR vitality[Title] OR resilience[Title] OR "quality of life"[Title] OR QoL[Title] OR autonomy[Title] OR competence[Title]) OR ("Psychological Well-Being"[MeSH Terms])) OR ("Quality of Life"[MeSH Terms])) OR ("heart rate"[MeSH Terms])) OR ("mental health"[MeSH Terms]) OR ((((((((((("social wellbeing"[Title] OR "social well-being"[Title] OR "social well being"[Title] OR "social capital"[Title] OR "social capacity"[Title] OR "social participation"[Title] OR "social functioning"[Title] OR "social actual*"[Title] OR "social inclusion"[Title] OR "social intel*"[Title] OR "social accept*"[Title] OR "social identi*"[Title] OR "social connect*"[Title] OR collective*[Title] OR psychosocial[Title] OR psycho-social[Title] OR collaboration[Title] OR affiliation*[Title] OR community[Title] OR cohesi*[Title] OR belong*[Title] OR benevolen*[Title] OR relat*[Title] OR "social support*"[Title] OR friendship*[Title] OR "connect* to others"[Title] OR prosocial[Title]) OR ("social status"[MeSH Terms])) OR ("social capital"[MeSH Terms])) OR ("social cohesion"[MeSH Terms])) OR ("social inclusion"[MeSH Terms])) OR ("social identification"[MeSH Terms])) OR ("Emotional Intelligence"[MeSH Terms])) OR ("social interaction"[MeSH Terms])) OR ("social participation"[MeSH Terms])) OR ("Interpersonal Relations"[MeSH Terms])) OR (Altruism[MeSH Terms]) OR ("planetary wellbeing"[Title] OR "planetary well being"[Title] OR "planetary well-being"[Title] OR "environmental wellbeing"[Title] OR "environmental well being"[Title] OR "environmental well-being"[Title] OR "environment* attitude*"[Title] OR "environment* concern*"[Title] OR "environment* belief*"[Title] OR "environment* behav*"[Title] OR "environment* friend*"[Title] OR "environment* protect*"[Title] OR "environment* preserv*"[Title] OR "environment* steward*"[Title] OR "environment* identity"[Title] OR "environment* interdepend*"[Title] OR "ecological wellbeing"[Title] OR "ecological well-being"[Title] OR "ecological well being"[Title] OR "ecolog* attitude"[Title] OR "eco attitude"[Title] OR "ecolog* behave*"[Title] OR "eco behav*"[Title] OR "ecolog* concern"[Title] OR "eco concern"[Title] OR "ecolog* belief"[Title] OR "eco belief"[Title] OR pro-environment*[Title] OR "pro environment*"[Title] OR "nature preserv*"[Title] OR "nature connect*"[Title] OR "nature relat*"[Title] OR "nature protect*"[Title] OR "nature in self"[Title] OR "nature behav*"[Title] OR "preservation behave*"[Title] OR "protection behav*"[Title] OR sustainab*[Title] OR biophil*[Title] OR "bio phil*"[Title] OR "climate friend*"[Title] OR "climate conservation"[Title] OR "climate * attitude*"[Title] OR "climate * behav*"[Title] OR "climate * belief*"[Title] OR "climate * aware*"[Title] OR "conservation* behav*"[Title] AND (("breathing intervention*"[Title] OR "breathing practice*"[Title] OR "breath* training"[Title] OR "breathing technique*"[Title] OR "breathing exercise*"[Title] OR "respiration practice"[Title] OR "breathing pattern"[Title] OR breathwork[Title] OR "breath* work"[Title] OR "control* respiration"[Title] OR "control* breath*"[Title] OR "diaphragm* breath*"[Title] OR "abdom* breath*"[Title] OR "thoracic breath*"[Title] OR "chest breath*"[Title] OR "clavicul* breath*"[Title] OR "rhythmic breath*"[Title] OR "functional breath*"[Title] OR "belly breath*"[Title] OR "deep breath*"[Title] OR "slow breath*"[Title] OR "fast breath*"[Title] OR "shallow breath*"[Title] OR "nostril breath*"[Title] OR "nose breath*"[Title] OR "mouth breath*"[Title] OR "pursed lip breath*"[Title] OR "pursed-lip breath*"[Title] OR "4 7 8 breath*"[Title] OR "4-7-8 breath*"[Title] OR "4 4 8 breath*"[Title] OR "4-4-8 breath*"[Title] OR "2-4 breath*"[Title] OR "five-finger breath*"[Title] OR "resonant breath*"[Title] OR "coherent breath*"[Title] OR "paced breath*"[Title] OR pranayama[Title] OR "yogic breath*"[Title] OR "yoga breath*"[Title] OR "sudarshan kriya yoga"[Title] OR kapalabhati[Title] OR "kapal bhati"[Title] OR "skull shining breath*"[Title] OR "nadi shodan*"[Title] OR "nadi shodhan*"[Title] OR "nadi sodhana"[Title] OR "alternate nostril breath*"[Title] OR "alternate nostril technique"[Title] OR "nadi sudi"[Title] OR "nadi suddhi"[Title] OR "nadi shuddhi"[Title] OR "anulom viloma"[Title] OR viloma[Title] OR "anulomviloma"[Title] OR "nerve purify* breath*"[Title] OR "channel cleaning breath*"[Title] OR ujjayi[Title] OR "ocean breath*"[Title] OR "victorious breath*"[Title] OR bhramari[Title] OR "bee breath*"[Title] OR "humming breath*"[Title] OR dirga[Title] OR dirgha[Title] OR "three part breath*"[Title] OR "three-part breath*"[Title] OR "circular breath*"[Title] OR "infant breath*"[Title] OR bhastrika[Title] OR "bellow* breath*"[Title] OR sitali[Title] OR sithali[Title] OR sheetali[Title] OR "cooling breath*"[Title] OR sheetkari[Title] OR sitkari[Title] OR "sipping breath*"[Title] OR "hissing breath*"[Title] OR samavritti[Title] OR "sama vritti"[Title] OR "square breath*"[Title] OR "equal breath*"[Title] OR "box breath*"[Title] OR plavini[Title] OR "floating breath*"[Title] OR "murcha breath*"[Title] OR "moorcha breath*"[Title] OR "swooning breath*"[Title] OR "surya bhedana"[Title] OR suryabhedana[Title] OR "sun piercing breath*"[Title] OR "surya nadi"[Title] OR "chandra bhedana"[Title] OR chandrabhedana[Title] OR "moon piercing breath*"[Title] OR "chandra nadi"[Title] OR "sukka purvaka"[Title] OR "shwasa prashwasa"[Title] OR "gita prana*"[Title] OR "sukha prana*"[Title] OR "easy* breath*"[Title] OR "nisshesha rechaka kumbhaka"[Title] OR shunyaka[Title] OR "bahya"[Title] OR "complete exhal*"[Title] OR "outer breath retention"[Title] OR "antara kumbhaka"[Title] OR "internal breath retention"[Title] OR "kevala kumbhaka"[Title] OR "absolute retention"[Title] OR "spontaneous retention"[Title] OR "complete retention"[Title] OR "breath of fire"[Title] OR "agni prana"[Title] OR "fire breath*"[Title] OR "simha* breath*"[Title] OR "lion* breath*"[Title] OR "qigong breath*"[Title] OR "wave breath*"[Title] OR "xingqi"[Title] OR "circulating breath*"[Title] OR "kidney breathing"[Title] OR "lower back breath*"[Title] OR "conscious* connected breath*"[Title] OR "quantum light breath*"[Title] OR "tummo"[Title] OR "pico pico breath*"[Title] OR "piko piko breath*"[Title] OR "soma breath*"[Title] OR "wim hof method"[Title] OR "oxygen advantage"[Title]) OR ("breathing exercises"[MeSH Terms]) |
|  | Health conditions | #7 | cancer[Title] OR patient*[Title] OR diabetes[Title] OR COPD[Title] OR asthma[Title] OR disease*[Title] OR cystic fibrosis[Title] OR myocardial infarction[Title] OR hypertension[Title] OR treatment[Title] |
|  | Holistic wellbeing AND breathing interventions NOT health conditions | #8 | #6 NOT #7 |
| APA | Individual wellbeing | S1 | TI (Wellbeing OR “well being” OR well-being OR flourish* OR self-connect* OR “connect* to self” OR “connect* with self” OR self-awareness OR self-accept* OR self-alignment OR “align* with self” OR self-determination OR self-efficacy OR self-esteem OR self-identity OR self-concept OR consciousness OR interoception OR “internal sensation*” OR “internal state*” OR intuition* OR “true self” OR “psychological* health” OR “emotional health” OR “mental health” OR “life satisfaction” OR “satisf* with life” OR “positive affect” OR “negative affect” OR “physical health” OR “positive emotion*” OR “negative emotion*” OR joy OR “heart rate variability” OR HRV OR hedoni* OR euda*moni* OR eudemon* OR happiness OR engag* OR meaning OR accomplishment OR achievement OR purpose OR “environmental mastery” OR “personal growth” OR matter* OR value* OR thriv* OR vitality OR resilience OR “quality of life” OR QoL OR autonomy OR competence) OR AB (Wellbeing OR “well being” OR well-being OR flourish* OR self-connect* OR “connect* to self” OR “connect* with self” OR self-awareness OR self-accept* OR self-alignment OR “align* with self” OR self-determination OR self-efficacy OR self-esteem OR self-identity OR self-concept OR consciousness OR interoception OR “internal sensation*” OR “internal state*” OR intuition* OR “true self” OR “psychological* health” OR “emotional health” OR “mental health” OR “life satisfaction” OR “satisf* with life” OR “positive affect” OR “negative affect” OR “physical health” OR “positive emotion*” OR “negative emotion*” OR joy OR “heart rate variability” OR HRV OR hedoni* OR euda*moni* OR eudemon* OR happiness OR engag* OR meaning OR accomplishment OR achievement OR purpose OR “environmental mastery” OR “personal growth” OR matter* OR value* OR thriv* OR vitality OR resilience OR “quality of life” OR QoL OR autonomy OR competence) OR DE (“well being+” OR “Self-acceptance” OR “self-determination” OR “self-efficacy” OR “self-esteem” OR “self-concept” OR “self-knowledge” OR “Self-management” OR “self-monitoring” OR “self-perception” OR “self-reference” OR “interoception” OR intuition OR “mental health+” OR “life satisfaction” OR “physical health” OR “holistic health” OR “positive emotions” OR “negative emotions” OR “heart rate+” OR happiness OR autonomy OR “quality of life” OR meaning OR meaningfulness OR achievement OR “sense of purpose” OR values) |
|  | Collective wellbeing | S2 | TI (“social wellbeing” OR “social well-being” OR “social well being” OR “social capital” OR “social capacity” OR “social participation” OR “social functioning” OR “social actual*” OR “social inclusion” OR “social intel*” OR “social accept*” OR “social identi*” OR “social connect*” OR collective* OR psychosocial OR psycho-social OR collaboration OR affiliation* OR community OR cohesi* OR belong* OR benevolen* OR relat* OR “social support*” OR friendship* OR “connect* to others” OR prosocial) OR AB (“social wellbeing” OR “social well-being” OR “social well being” OR “social capital” OR “social capacity” OR “social participation” OR “social functioning” OR “social actual*” OR “social inclusion” OR “social intel*” OR “social accept*” OR “social identi*” OR “social connect*” OR collective* OR psychosocial OR psycho-social OR collaboration OR affiliation* OR community OR cohesi* OR belong* OR benevolen* OR relat* OR “social support*” OR friendship* OR “connect* to others” OR prosocial) OR DE (“social well being” OR “relationship quality” OR “social resources” OR “psychosocial outcomes” OR “psychosocial factors” OR “interpersonal interaction” OR “social interaction” OR “interpersonal relationships+” OR “relationship satisfaction+” OR “relationship quality” OR “social identity” OR “group identity” OR “social acceptance+” OR “social capital” OR “social cohesion” OR “group cohesion” OR “affiliation motivation” OR “social connectedness” OR “social inclusion” OR “social integration” OR “social isolation” OR “social networks” OR “social support+” OR “social health” OR “sense of community” OR “social functioning” OR “group participation” OR “social groups” OR “community health” OR “community mental health” OR “population health” OR “global mental health” OR “community involvement” OR collaboration OR “prosocial behavior+”) |
|  | Planetary wellbeing | S3 | TI (“planetary wellbeing” OR “planetary well being” OR “planetary well-being” OR “environmental wellbeing” OR “environmental well being” OR “environmental well-being” OR “environment* attitude*” OR “environment* concern*” OR “environment* belief*” OR “environment* behav*” OR “environment* friend*” OR “environment* protect*” OR “environment* preserv*” OR “environment* steward*” OR “environment* identity” OR “environment* interdepend*” OR “ecological wellbeing” OR “ecological well-being” OR “ecological well being” OR “ecolog* attitude” OR “eco attitude” OR “ecolog* behave*” OR “eco behav*” OR “ecolog* concern” OR “eco concern” OR “ecolog* belief” OR “eco belief” OR pro-environment* OR “pro environment*” OR “nature preserv*” OR “nature connect*” OR “nature relat*” OR “nature protect*” OR “nature in self” OR “nature behav*” OR “preservation behave*” OR “protection behav*” OR sustainab* OR biophil* OR “bio phil*” OR “climate friend*” OR “climate conservation” OR “climate * attitude*” OR “climate * behav*” OR “climate * belief*” OR “climate * aware*” OR “conservation* behav*”) OR AB (“planetary wellbeing” OR “planetary well being” OR “planetary well-being” OR “environmental wellbeing” OR “environmental well being” OR “environmental well-being” OR “environment* attitude*” OR “environment* concern*” OR “environment* belief*” OR “environment* behav*” OR “environment* friend*” OR “environment* protect*” OR “environment* preserv*” OR “environment* steward*” OR “environment* identity” OR “environment* interdepend*” OR “ecological wellbeing” OR “ecological well-being” OR “ecological well being” OR “ecolog* attitude” OR “eco attitude” OR “ecolog* behave*” OR “eco behav*” OR “ecolog* concern” OR “eco concern” OR “ecolog* belief” OR “eco belief” OR pro-environment* OR “pro environment*” OR “nature preserv*” OR “nature connect*” OR “nature relat*” OR “nature protect*” OR “nature in self” OR “nature behav*” OR “preservation behave*” OR “protection behav*” OR sustainab* OR biophil* OR “bio phil*” OR “climate friend*” OR “climate conservation” OR “climate * attitude*” OR “climate * behav*” OR “climate * belief*” OR “climate * aware*” OR “conservation* behav*”) OR DE (“nature (environment)+” OR “pro environmental behavior+” OR “environmental sustainability” OR “climate change+” OR “environmental attitudes+” OR “ecological factors+” OR “environmental effects”) |
|  | Holistic wellbeing | S4 | S1 OR S2 OR S3 |
|  | Breathing interventions | S5 | TI (“breathing intervention*” OR “breathing practice*” OR “breath* training” OR “breathing technique*” OR “breathing exercise*” OR “respiration practice” OR “breathing pattern” OR breathwork OR “breath* work” OR “control* respiration” OR “control* breath*” OR “diaphragm* breath*” OR “abdom* breath*” OR “thoracic breath*” OR “chest breath*” OR “clavicul* breath*” OR “rhythmic breath*” OR “functional breath*” OR “belly breath*” OR “deep breath*” OR “slow breath*” OR “fast breath*” OR “shallow breath*” OR “nostril breath*” OR “nose breath*” OR “mouth breath*” OR “pursed lip breath*” OR “pursed-lip breath*” OR “4 7 8 breath*” OR “4-7-8 breath*” OR “4 4 8 breath*” OR “4-4-8 breath*” OR “2-4 breath*” OR “five-finger breath*” OR “resonant breath*” OR “coherent breath*” OR “paced breath*” OR pranayama OR “yogic breath*” OR “yoga breath*” OR “sudarshan kriya yoga” OR kapalabhati OR “kapal bhati” OR “skull shining breath*” OR “nadi shodan*” OR “nadi shodhan*” OR “nadi sodhana” OR “alternate nostril breath*” OR “alternate nostril technique” OR “nadi sudi” OR “nadi suddhi” OR “nadi shuddhi” OR “anulom viloma” OR viloma OR “anulomviloma” OR “nerve purify* breath*” OR “channel cleaning breath*” OR ujjayi OR “ocean breath*” OR “victorious breath*” OR bhramari OR “bee breath*” OR “humming breath*” OR dirga OR dirgha OR “three part breath*” OR “three-part breath*” OR “circular breath*” OR “infant breath*” OR bhastrika OR “bellow* breath*” OR sitali OR sithali OR sheetali OR “cooling breath*” OR sheetkari OR sitkari OR “sipping breath*” OR “hissing breath*” OR samavritti OR “sama vritti” OR “square breath*” OR “equal breath*” OR “box breath*” OR plavini OR “floating breath*” OR “murcha breath*” OR “moorcha breath*” OR “swooning breath*” OR “surya bhedana” OR suryabhedana OR “sun piercing breath*” OR “surya nadi” OR “chandra bhedana” OR chandrabhedana OR “moon piercing breath*” OR “chandra nadi” OR “sukka purvaka” OR “shwasa prashwasa” OR “gita prana*” OR “sukha prana*” OR “easy* breath*” OR “nisshesha rechaka kumbhaka” OR shunyaka OR “bahya” OR “complete exhal*” OR “outer breath retention” OR “antara kumbhaka” OR “internal breath retention” OR “kevala kumbhaka” OR “absolute retention” OR “spontaneous retention” OR “complete retention” OR “breath of fire” OR “agni prana” OR “fire breath*” OR “simha* breath*” OR “lion* breath*” OR “qigong breath*” OR “wave breath*” OR “xingqi” OR “circulating breath*” OR “kidney breathing” OR “lower back breath*” OR “conscious* connected breath*” OR “quantum light breath*” OR “tummo” OR “pico pico breath*” OR “piko piko breath*” OR “soma breath*” OR “wim hof method” OR “oxygen advantage”) OR AB (“breathing intervention*” OR “breathing practice*” OR “breath* training” OR “breathing technique*” OR “breathing exercise*” OR “respiration practice” OR “breathing pattern” OR breathwork OR “breath* work” OR “control* respiration” OR “control* breath*” OR “diaphragm* breath*” OR “abdom* breath*” OR “thoracic breath*” OR “chest breath*” OR “clavicul* breath*” OR “rhythmic breath*” OR “functional breath*” OR “belly breath*” OR “deep breath*” OR “slow breath*” OR “fast breath*” OR “shallow breath*” OR “nostril breath*” OR “nose breath*” OR “mouth breath*” OR “pursed lip breath*” OR “pursed-lip breath*” OR “4 7 8 breath*” OR “4-7-8 breath*” OR “4 4 8 breath*” OR “4-4-8 breath*” OR “2-4 breath*” OR “five-finger breath*” OR “resonant breath*” OR “coherent breath*” OR “paced breath*” OR pranayama OR “yogic breath*” OR “yoga breath*” OR “sudarshan kriya yoga” OR kapalabhati OR “kapal bhati” OR “skull shining breath*” OR “nadi shodan*” OR “nadi shodhan*” OR “nadi sodhana” OR “alternate nostril breath*” OR “alternate nostril technique” OR “nadi sudi” OR “nadi suddhi” OR “nadi shuddhi” OR “anulom viloma” OR viloma OR “anulomviloma” OR “nerve purify* breath*” OR “channel cleaning breath*” OR ujjayi OR “ocean breath*” OR “victorious breath*” OR bhramari OR “bee breath*” OR “humming breath*” OR dirga OR dirgha OR “three part breath*” OR “three-part breath*” OR “circular breath*” OR “infant breath*” OR bhastrika OR “bellow* breath*” OR sitali OR sithali OR sheetali OR “cooling breath*” OR sheetkari OR sitkari OR “sipping breath*” OR “hissing breath*” OR samavritti OR “sama vritti” OR “square breath*” OR “equal breath*” OR “box breath*” OR plavini OR “floating breath*” OR “murcha breath*” OR “moorcha breath*” OR “swooning breath*” OR “surya bhedana” OR suryabhedana OR “sun piercing breath*” OR “surya nadi” OR “chandra bhedana” OR chandrabhedana OR “moon piercing breath*” OR “chandra nadi” OR “sukka purvaka” OR “shwasa prashwasa” OR “gita prana*” OR “sukha prana*” OR “easy* breath*” OR “nisshesha rechaka kumbhaka” OR shunyaka OR “bahya” OR “complete exhal*” OR “outer breath retention” OR “antara kumbhaka” OR “internal breath retention” OR “kevala kumbhaka” OR “absolute retention” OR “spontaneous retention” OR “complete retention” OR “breath of fire” OR “agni prana” OR “fire breath*” OR “simha* breath*” OR “lion* breath*” OR “qigong breath*” OR “wave breath*” OR “xingqi” OR “circulating breath*” OR “kidney breathing” OR “lower back breath*” OR “conscious* connected breath*” OR “quantum light breath*” OR “tummo” OR “pico pico breath*” OR “piko piko breath*” OR “soma breath*” OR “wim hof method” OR “oxygen advantage”) OR DE “breathing techniques” |
|  | Holistic wellbeing AND breathing interventions | S6 | S4 AND S5 |
|  | Health conditions | S7 | TI (cancer OR patient* OR diabetes OR COPD OR asthma OR disease* OR cystic fibrosis OR myocardial infarction OR hypertension OR treatment) OR AB (cancer OR patient* OR diabetes OR COPD OR asthma OR disease* OR cystic fibrosis OR myocardial infarction OR hypertension OR treatment) |
|  | Holistic wellbeing AND breathing interventions NOT health conditions | S8 | S6 NOT S7 |
| Scopus | Individual wellbeing | #1 | TITLE ( wellbeing OR "well being" OR well-being OR flourish* OR self-connect* OR "connect* to self" OR "connect* with self" OR self-awareness OR self-accept* OR self-alignment OR "align* with self" OR self-determination OR self-efficacy OR self-esteem OR self-identity OR self-concept OR consciousness OR interoception OR "internal sensation*" OR "internal state*" OR intuition* OR "true self" OR "psychological* health" OR "emotional health" OR "mental health" OR "life satisfaction" OR "satisf* with life" OR "positive affect" OR "negative affect" OR "physical health" OR "positive emotion*" OR "negative emotion*" OR joy OR "heart rate variability" OR hrv OR hedoni* OR euda*moni* OR eudemon* OR happiness OR engag* OR meaning OR accomplishment OR achievement OR purpose OR "environmental mastery" OR "personal growth" OR matter* OR value* OR thriv* OR vitality OR resilience OR "quality of life" OR qol OR autonomy OR competence ) |
|  | Collective wellbeing | #2 | TITLE ( "social wellbeing" OR "social well-being" OR "social well being" OR "social capital" OR "social capacity" OR "social participation" OR "social functioning" OR "social actual*" OR "social inclusion" OR "social intel*" OR "social accept*" OR "social identi*" OR "social connect*" OR collective* OR psychosocial OR psycho-social OR collaboration OR affiliation* OR community OR cohesi* OR belong* OR benevolen* OR relat* OR "social support*" OR friendship* OR "connect* to others" OR prosocial ) |
|  | Planetary wellbeing | #3 | TITLE ( "planetary wellbeing" OR "planetary well being" OR "planetary well-being" OR "environmental wellbeing" OR "environmental well being" OR "environmental well-being" OR "environment* attitude*" OR "environment* concern*" OR "environment* belief*" OR "environment* behav*" OR "environment* friend*" OR "environment* protect*" OR "environment* preserv*" OR "environment* steward*" OR "environment* identity" OR "environment* interdepend*" OR "ecological wellbeing" OR "ecological well-being" OR "ecological well being" OR "ecolog* attitude" OR "eco attitude" OR "ecolog* behave*" OR "eco behav*" OR "ecolog* concern" OR "eco concern" OR "ecolog* belief" OR "eco belief" OR pro-environment* OR "pro environment*" OR "nature preserv*" OR "nature connect*" OR "nature relat*" OR "nature protect*" OR "nature in self" OR "nature behav*" OR "preservation behave*" OR "protection behav*" OR sustainab* OR biophil* OR "bio phil*" OR "climate friend*" OR "climate conservation" OR "climate * attitude*" OR "climate * behav*" OR "climate * belief*" OR "climate * aware*" OR "conservation* behav*" ) |
|  | Holistic wellbeing | #4 | #1 OR #2 OR #3  ( TITLE ( wellbeing OR "well being" OR well-being OR flourish* OR self-connect* OR "connect* to self" OR "connect* with self" OR self-awareness OR self-accept* OR self-alignment OR "align* with self" OR self-determination OR self-efficacy OR self-esteem OR self-identity OR self-concept OR consciousness OR interoception OR "internal sensation*" OR "internal state*" OR intuition* OR "true self" OR "psychological* health" OR "emotional health" OR "mental health" OR "life satisfaction" OR "satisf* with life" OR "positive affect" OR "negative affect" OR "physical health" OR "positive emotion*" OR "negative emotion*" OR joy OR "heart rate variability" OR hrv OR hedoni* OR euda*moni* OR eudemon* OR happiness OR engag* OR meaning OR accomplishment OR achievement OR purpose OR "environmental mastery" OR "personal growth" OR matter* OR value* OR thriv* OR vitality OR resilience OR "quality of life" OR qol OR autonomy OR competence ) ) OR ( TITLE ( "social wellbeing" OR "social well-being" OR "social well being" OR "social capital" OR "social capacity" OR "social participation" OR "social functioning" OR "social actual*" OR "social inclusion" OR "social intel*" OR "social accept*" OR "social identi*" OR "social connect*" OR collective* OR psychosocial OR psycho-social OR collaboration OR affiliation* OR community OR cohesi* OR belong* OR benevolen* OR relat* OR "social support*" OR friendship* OR "connect* to others" OR prosocial ) ) OR ( TITLE ( "planetary wellbeing" OR "planetary well being" OR "planetary well-being" OR "environmental wellbeing" OR "environmental well being" OR "environmental well-being" OR "environment* attitude*" OR "environment* concern*" OR "environment* belief*" OR "environment* behav*" OR "environment* friend*" OR "environment* protect*" OR "environment* preserv*" OR "environment* steward*" OR "environment* identity" OR "environment* interdepend*" OR "ecological wellbeing" OR "ecological well-being" OR "ecological well being" OR "ecolog* attitude" OR "eco attitude" OR "ecolog* behave*" OR "eco behav*" OR "ecolog* concern" OR "eco concern" OR "ecolog* belief" OR "eco belief" OR pro-environment* OR "pro environment*" OR "nature preserv*" OR "nature connect*" OR "nature relat*" OR "nature protect*" OR "nature in self" OR "nature behav*" OR "preservation behave*" OR "protection behav*" OR sustainab* OR biophil* OR "bio phil*" OR "climate friend*" OR "climate conservation" OR "climate * attitude*" OR "climate * behav*" OR "climate * belief*" OR "climate * aware*" OR "conservation* behav*" ) ) |
|  | Breathing interventions | #5 | TITLE ( "breathing intervention*" OR "breathing practice*" OR "breath* training" OR "breathing technique*" OR "breathing exercise*" OR "respiration practice" OR "breathing pattern" OR breathwork OR "breath* work" OR "control* respiration" OR "control* breath*" OR "diaphragm* breath*" OR "abdom* breath*" OR "thoracic breath*" OR "chest breath*" OR "clavicul* breath*" OR "rhythmic breath*" OR "functional breath*" OR "belly breath*" OR "deep breath*" OR "slow breath*" OR "fast breath*" OR "shallow breath*" OR "nostril breath*" OR "nose breath*" OR "mouth breath*" OR "pursed lip breath*" OR "pursed-lip breath*" OR "4 7 8 breath*" OR "4-7-8 breath*" OR "4 4 8 breath*" OR "4-4-8 breath*" OR "2-4 breath*" OR "five-finger breath*" OR "resonant breath*" OR "coherent breath*" OR "paced breath*" OR pranayama OR "yogic breath*" OR "yoga breath*" OR "sudarshan kriya yoga" OR kapalabhati OR "kapal bhati" OR "skull shining breath*" OR "nadi shodan*" OR "nadi shodhan*" OR "nadi sodhana" OR "alternate nostril breath*" OR "alternate nostril technique" OR "nadi sudi" OR "nadi suddhi" OR "nadi shuddhi" OR "anulom viloma" OR viloma OR "anulomviloma" OR "nerve purify* breath*" OR "channel cleaning breath*" OR ujjayi OR "ocean breath*" OR "victorious breath*" OR bhramari OR "bee breath*" OR "humming breath*" OR dirga OR dirgha OR "three part breath*" OR "three-part breath*" OR "circular breath*" OR "infant breath*" OR bhastrika OR "bellow* breath*" OR sitali OR sithali OR sheetali OR "cooling breath*" OR sheetkari OR sitkari OR "sipping breath*" OR "hissing breath*" OR samavritti OR "sama vritti" OR "square breath*" OR "equal breath*" OR "box breath*" OR plavini OR "floating breath*" OR "murcha breath*" OR "moorcha breath*" OR "swooning breath*" OR "surya bhedana" OR suryabhedana OR "sun piercing breath*" OR "surya nadi" OR "chandra bhedana" OR chandrabhedana OR "moon piercing breath*" OR "chandra nadi" OR "sukka purvaka" OR "shwasa prashwasa" OR "gita prana*" OR "sukha prana*" OR "easy* breath*" OR "nisshesha rechaka kumbhaka" OR shunyaka OR "bahya" OR "complete exhal*" OR "outer breath retention" OR "antara kumbhaka" OR "internal breath retention" OR "kevala kumbhaka" OR "absolute retention" OR "spontaneous retention" OR "complete retention" OR "breath of fire" OR "agni prana" OR "fire breath*" OR "simha* breath*" OR "lion* breath*" OR "qigong breath*" OR "wave breath*" OR "xingqi" OR "circulating breath*" OR "kidney breathing" OR "lower back breath*" OR "conscious* connected breath*" OR "quantum light breath*" OR "tummo" OR "pico pico breath*" OR "piko piko breath*" OR "soma breath*" OR "wim hof method" OR "oxygen advantage" ) |
|  | Holistic wellbeing AND breathing interventions | #6 | #4 AND #5  ( ( TITLE ( wellbeing OR "well being" OR well-being OR flourish* OR self-connect* OR "connect* to self" OR "connect* with self" OR self-awareness OR self-accept* OR self-alignment OR "align* with self" OR self-determination OR self-efficacy OR self-esteem OR self-identity OR self-concept OR consciousness OR interoception OR "internal sensation*" OR "internal state*" OR intuition* OR "true self" OR "psychological* health" OR "emotional health" OR "mental health" OR "life satisfaction" OR "satisf* with life" OR "positive affect" OR "negative affect" OR "physical health" OR "positive emotion*" OR "negative emotion*" OR joy OR "heart rate variability" OR hrv OR hedoni* OR euda*moni* OR eudemon* OR happiness OR engag* OR meaning OR accomplishment OR achievement OR purpose OR "environmental mastery" OR "personal growth" OR matter* OR value* OR thriv* OR vitality OR resilience OR "quality of life" OR qol OR autonomy OR competence ) ) OR ( TITLE ( "social wellbeing" OR "social well-being" OR "social well being" OR "social capital" OR "social capacity" OR "social participation" OR "social functioning" OR "social actual*" OR "social inclusion" OR "social intel*" OR "social accept*" OR "social identi*" OR "social connect*" OR collective* OR psychosocial OR psycho-social OR collaboration OR affiliation* OR community OR cohesi* OR belong* OR benevolen* OR relat* OR "social support*" OR friendship* OR "connect* to others" OR prosocial ) ) OR ( TITLE ( "planetary wellbeing" OR "planetary well being" OR "planetary well-being" OR "environmental wellbeing" OR "environmental well being" OR "environmental well-being" OR "environment* attitude*" OR "environment* concern*" OR "environment* belief*" OR "environment* behav*" OR "environment* friend*" OR "environment* protect*" OR "environment* preserv*" OR "environment* steward*" OR "environment* identity" OR "environment* interdepend*" OR "ecological wellbeing" OR "ecological well-being" OR "ecological well being" OR "ecolog* attitude" OR "eco attitude" OR "ecolog* behave*" OR "eco behav*" OR "ecolog* concern" OR "eco concern" OR "ecolog* belief" OR "eco belief" OR pro-environment* OR "pro environment*" OR "nature preserv*" OR "nature connect*" OR "nature relat*" OR "nature protect*" OR "nature in self" OR "nature behav*" OR "preservation behave*" OR "protection behav*" OR sustainab* OR biophil* OR "bio phil*" OR "climate friend*" OR "climate conservation" OR "climate * attitude*" OR "climate * behav*" OR "climate * belief*" OR "climate * aware*" OR "conservation* behav*" ) ) ) AND ( TITLE ( "breathing intervention*" OR "breathing practice*" OR "breath* training" OR "breathing technique*" OR "breathing exercise*" OR "respiration practice" OR "breathing pattern" OR breathwork OR "breath* work" OR "control* respiration" OR "control* breath*" OR "diaphragm* breath*" OR "abdom* breath*" OR "thoracic breath*" OR "chest breath*" OR "clavicul* breath*" OR "rhythmic breath*" OR "functional breath*" OR "belly breath*" OR "deep breath*" OR "slow breath*" OR "fast breath*" OR "shallow breath*" OR "nostril breath*" OR "nose breath*" OR "mouth breath*" OR "pursed lip breath*" OR "pursed-lip breath*" OR "4 7 8 breath*" OR "4-7-8 breath*" OR "4 4 8 breath*" OR "4-4-8 breath*" OR "2-4 breath*" OR "five-finger breath*" OR "resonant breath*" OR "coherent breath*" OR "paced breath*" OR pranayama OR "yogic breath*" OR "yoga breath*" OR "sudarshan kriya yoga" OR kapalabhati OR "kapal bhati" OR "skull shining breath*" OR "nadi shodan*" OR "nadi shodhan*" OR "nadi sodhana" OR "alternate nostril breath*" OR "alternate nostril technique" OR "nadi sudi" OR "nadi suddhi" OR "nadi shuddhi" OR "anulom viloma" OR viloma OR "anulomviloma" OR "nerve purify* breath*" OR "channel cleaning breath*" OR ujjayi OR "ocean breath*" OR "victorious breath*" OR bhramari OR "bee breath*" OR "humming breath*" OR dirga OR dirgha OR "three part breath*" OR "three-part breath*" OR "circular breath*" OR "infant breath*" OR bhastrika OR "bellow* breath*" OR sitali OR sithali OR sheetali OR "cooling breath*" OR sheetkari OR sitkari OR "sipping breath*" OR "hissing breath*" OR samavritti OR "sama vritti" OR "square breath*" OR "equal breath*" OR "box breath*" OR plavini OR "floating breath*" OR "murcha breath*" OR "moorcha breath*" OR "swooning breath*" OR "surya bhedana" OR suryabhedana OR "sun piercing breath*" OR "surya nadi" OR "chandra bhedana" OR chandrabhedana OR "moon piercing breath*" OR "chandra nadi" OR "sukka purvaka" OR "shwasa prashwasa" OR "gita prana*" OR "sukha prana*" OR "easy* breath*" OR "nisshesha rechaka kumbhaka" OR shunyaka OR "bahya" OR "complete exhal*" OR "outer breath retention" OR "antara kumbhaka" OR "internal breath retention" OR "kevala kumbhaka" OR "absolute retention" OR "spontaneous retention" OR "complete retention" OR "breath of fire" OR "agni prana" OR "fire breath*" OR "simha* breath*" OR "lion* breath*" OR "qigong breath*" OR "wave breath*" OR "xingqi" OR "circulating breath*" OR "kidney breathing" OR "lower back breath*" OR "conscious* connected breath*" OR "quantum light breath*" OR "tummo" OR "pico pico breath*" OR "piko piko breath*" OR "soma breath*" OR "wim hof method" OR "oxygen advantage" ) ) |
|  | Health conditions | #7 | TITLE ( cancer OR patient* OR diabetes OR copd OR asthma OR disease* OR cystic AND fibrosis OR myocardial AND infarction OR hypertension OR treatment ) |
|  | Holistic wellbeing AND breathing interventions  NOT breathing interventions | #8 | #6 NOT #7  ( ( ( TITLE ( wellbeing OR "well being" OR well-being OR flourish* OR self-connect* OR "connect* to self" OR "connect* with self" OR self-awareness OR self-accept* OR self-alignment OR "align* with self" OR self-determination OR self-efficacy OR self-esteem OR self-identity OR self-concept OR consciousness OR interoception OR "internal sensation*" OR "internal state*" OR intuition* OR "true self" OR "psychological* health" OR "emotional health" OR "mental health" OR "life satisfaction" OR "satisf* with life" OR "positive affect" OR "negative affect" OR "physical health" OR "positive emotion*" OR "negative emotion*" OR joy OR "heart rate variability" OR hrv OR hedoni* OR euda*moni* OR eudemon* OR happiness OR engag* OR meaning OR accomplishment OR achievement OR purpose OR "environmental mastery" OR "personal growth" OR matter* OR value* OR thriv* OR vitality OR resilience OR "quality of life" OR qol OR autonomy OR competence ) ) OR ( TITLE ( "social wellbeing" OR "social well-being" OR "social well being" OR "social capital" OR "social capacity" OR "social participation" OR "social functioning" OR "social actual*" OR "social inclusion" OR "social intel*" OR "social accept*" OR "social identi*" OR "social connect*" OR collective* OR psychosocial OR psycho-social OR collaboration OR affiliation* OR community OR cohesi* OR belong* OR benevolen* OR relat* OR "social support*" OR friendship* OR "connect* to others" OR prosocial ) ) OR ( TITLE ( "planetary wellbeing" OR "planetary well being" OR "planetary well-being" OR "environmental wellbeing" OR "environmental well being" OR "environmental well-being" OR "environment* attitude*" OR "environment* concern*" OR "environment* belief*" OR "environment* behav*" OR "environment* friend*" OR "environment* protect*" OR "environment* preserv*" OR "environment* steward*" OR "environment* identity" OR "environment* interdepend*" OR "ecological wellbeing" OR "ecological well-being" OR "ecological well being" OR "ecolog* attitude" OR "eco attitude" OR "ecolog* behave*" OR "eco behav*" OR "ecolog* concern" OR "eco concern" OR "ecolog* belief" OR "eco belief" OR pro-environment* OR "pro environment*" OR "nature preserv*" OR "nature connect*" OR "nature relat*" OR "nature protect*" OR "nature in self" OR "nature behav*" OR "preservation behave*" OR "protection behav*" OR sustainab* OR biophil* OR "bio phil*" OR "climate friend*" OR "climate conservation" OR "climate * attitude*" OR "climate * behav*" OR "climate * belief*" OR "climate * aware*" OR "conservation* behav*" ) ) ) AND ( TITLE ( "breathing intervention*" OR "breathing practice*" OR "breath* training" OR "breathing technique*" OR "breathing exercise*" OR "respiration practice" OR "breathing pattern" OR breathwork OR "breath* work" OR "control* respiration" OR "control* breath*" OR "diaphragm* breath*" OR "abdom* breath*" OR "thoracic breath*" OR "chest breath*" OR "clavicul* breath*" OR "rhythmic breath*" OR "functional breath*" OR "belly breath*" OR "deep breath*" OR "slow breath*" OR "fast breath*" OR "shallow breath*" OR "nostril breath*" OR "nose breath*" OR "mouth breath*" OR "pursed lip breath*" OR "pursed-lip breath*" OR "4 7 8 breath*" OR "4-7-8 breath*" OR "4 4 8 breath*" OR "4-4-8 breath*" OR "2-4 breath*" OR "five-finger breath*" OR "resonant breath*" OR "coherent breath*" OR "paced breath*" OR pranayama OR "yogic breath*" OR "yoga breath*" OR "sudarshan kriya yoga" OR kapalabhati OR "kapal bhati" OR "skull shining breath*" OR "nadi shodan*" OR "nadi shodhan*" OR "nadi sodhana" OR "alternate nostril breath*" OR "alternate nostril technique" OR "nadi sudi" OR "nadi suddhi" OR "nadi shuddhi" OR "anulom viloma" OR viloma OR "anulomviloma" OR "nerve purify* breath*" OR "channel cleaning breath*" OR ujjayi OR "ocean breath*" OR "victorious breath*" OR bhramari OR "bee breath*" OR "humming breath*" OR dirga OR dirgha OR "three part breath*" OR "three-part breath*" OR "circular breath*" OR "infant breath*" OR bhastrika OR "bellow* breath*" OR sitali OR sithali OR sheetali OR "cooling breath*" OR sheetkari OR sitkari OR "sipping breath*" OR "hissing breath*" OR samavritti OR "sama vritti" OR "square breath*" OR "equal breath*" OR "box breath*" OR plavini OR "floating breath*" OR "murcha breath*" OR "moorcha breath*" OR "swooning breath*" OR "surya bhedana" OR suryabhedana OR "sun piercing breath*" OR "surya nadi" OR "chandra bhedana" OR chandrabhedana OR "moon piercing breath*" OR "chandra nadi" OR "sukka purvaka" OR "shwasa prashwasa" OR "gita prana*" OR "sukha prana*" OR "easy* breath*" OR "nisshesha rechaka kumbhaka" OR shunyaka OR "bahya" OR "complete exhal*" OR "outer breath retention" OR "antara kumbhaka" OR "internal breath retention" OR "kevala kumbhaka" OR "absolute retention" OR "spontaneous retention" OR "complete retention" OR "breath of fire" OR "agni prana" OR "fire breath*" OR "simha* breath*" OR "lion* breath*" OR "qigong breath*" OR "wave breath*" OR "xingqi" OR "circulating breath*" OR "kidney breathing" OR "lower back breath*" OR "conscious* connected breath*" OR "quantum light breath*" OR "tummo" OR "pico pico breath*" OR "piko piko breath*" OR "soma breath*" OR "wim hof method" OR "oxygen advantage" ) ) ) AND NOT ( TITLE ( cancer OR patient* OR diabetes OR copd OR asthma OR disease* OR cystic AND fibrosis OR myocardial AND infarction OR hypertension OR treatment ) ) |
| Web of Science | Individual wellbeing | #1 | (TI=(Wellbeing OR “well being” OR well-being OR flourish* OR self-connect* OR “connect* to self” OR “connect* with self” OR self-awareness OR self-accept* OR self-alignment OR “align* with self” OR self-determination OR self-efficacy OR self-esteem OR self-identity OR self-concept OR consciousness OR interoception OR “internal sensation*” OR “internal state*” OR intuition* OR “true self” OR “psychological* health” OR “emotional health” OR “mental health” OR “life satisfaction” OR “satisf* with life” OR “positive affect” OR “negative affect” OR “physical health” OR “positive emotion*” OR “negative emotion*” OR joy OR “heart rate variability” OR HRV OR hedoni* OR euda*moni* OR eudemon* OR happiness OR engag* OR meaning OR accomplishment OR achievement OR purpose OR “environmental mastery” OR “personal growth” OR matter* OR value* OR thriv* OR vitality OR resilience OR “quality of life” OR QoL OR autonomy OR competence)) |
|  | Collective wellbeing | #2 | (TI=(“social wellbeing” OR “social well-being” OR “social well being” OR “social capital” OR “social capacity” OR “social participation” OR “social functioning” OR “social actual*” OR “social inclusion” OR “social intel*” OR “social accept*” OR “social identi*” OR “social connect*” OR collective* OR psychosocial OR psycho-social OR collaboration OR affiliation* OR community OR cohesi* OR belong* OR benevolen* OR relat* OR “social support*” OR friendship* OR “connect* to others” OR prosocial)) |
|  | Planetary wellbeing | #3 | (TI=(“planetary wellbeing” OR “planetary well being” OR “planetary well-being” OR “environmental wellbeing” OR “environmental well being” OR “environmental well-being” OR “environment* attitude*” OR “environment* concern*” OR “environment* belief*” OR “environment* behav*” OR “environment* friend*” OR “environment* protect*” OR “environment* preserv*” OR “environment* steward*” OR “environment* identity” OR “environment* interdepend*” OR “ecological wellbeing” OR “ecological well-being” OR “ecological well being” OR “ecolog* attitude” OR “eco attitude” OR “ecolog* behave*” OR “eco behav*” OR “ecolog* concern” OR “eco concern” OR “ecolog* belief” OR “eco belief” OR pro-environment* OR “pro environment*” OR “nature preserv*” OR “nature connect*” OR “nature relat*” OR “nature protect*” OR “nature in self” OR “nature behav*” OR “preservation behave*” OR “protection behav*” OR sustainab* OR biophil* OR “bio phil*” OR “climate friend*” OR “climate conservation” OR “climate * attitude*” OR “climate * behav*” OR “climate * belief*” OR “climate * aware*” OR “conservation* behav*” )) |
|  | Holistic wellbeing | #4 | #1 OR #2 OR #3 |
|  | Breathing interventions | #5 | (TI=(“breathing intervention*” OR “breathing practice*” OR “breath* training” OR “breathing technique*” OR “breathing exercise*” OR “respiration practice” OR “breathing pattern” OR breathwork OR “breath* work” OR “control* respiration” OR “control* breath*” OR “diaphragm* breath*” OR “abdom* breath*” OR “thoracic breath*” OR “chest breath*” OR “clavicul* breath*” OR “rhythmic breath*” OR “functional breath*” OR “belly breath*” OR “deep breath*” OR “slow breath*” OR “fast breath*” OR “shallow breath*” OR “nostril breath*” OR “nose breath*” OR “mouth breath*” OR “pursed lip breath*” OR “pursed-lip breath*” OR “4 7 8 breath*” OR “4-7-8 breath*” OR “4 4 8 breath*” OR “4-4-8 breath*” OR “2-4 breath*” OR “five-finger breath*” OR “resonant breath*” OR “coherent breath*” OR “paced breath*” OR pranayama OR “yogic breath*” OR “yoga breath*” OR “sudarshan kriya yoga” OR kapalabhati OR “kapal bhati” OR “skull shining breath*” OR “nadi shodan*” OR “nadi shodhan*” OR “nadi sodhana” OR “alternate nostril breath*” OR “alternate nostril technique” OR “nadi sudi” OR “nadi suddhi” OR “nadi shuddhi” OR “anulom viloma” OR viloma OR “anulomviloma” OR “nerve purify* breath*” OR “channel cleaning breath*” OR ujjayi OR “ocean breath*” OR “victorious breath*” OR bhramari OR “bee breath*” OR “humming breath*” OR dirga OR dirgha OR “three part breath*” OR “three-part breath*” OR “circular breath*” OR “infant breath*” OR bhastrika OR “bellow* breath*” OR sitali OR sithali OR sheetali OR “cooling breath*” OR sheetkari OR sitkari OR “sipping breath*” OR “hissing breath*” OR samavritti OR “sama vritti” OR “square breath*” OR “equal breath*” OR “box breath*” OR plavini OR “floating breath*” OR “murcha breath*” OR “moorcha breath*” OR “swooning breath*” OR “surya bhedana” OR suryabhedana OR “sun piercing breath*” OR “surya nadi” OR “chandra bhedana” OR chandrabhedana OR “moon piercing breath*” OR “chandra nadi” OR “sukka purvaka” OR “shwasa prashwasa” OR “gita prana*” OR “sukha prana*” OR “easy* breath*” OR “nisshesha rechaka kumbhaka” OR shunyaka OR “bahya” OR “complete exhal*” OR “outer breath retention” OR “antara kumbhaka” OR “internal breath retention” OR “kevala kumbhaka” OR “absolute retention” OR “spontaneous retention” OR “complete retention” OR “breath of fire” OR “agni prana” OR “fire breath*” OR “simha* breath*” OR “lion* breath*” OR “qigong breath*” OR “wave breath*” OR “xingqi” OR “circulating breath*” OR “kidney breathing” OR “lower back breath*” OR “conscious* connected breath*” OR “quantum light breath*” OR “tummo” OR “pico pico breath*” OR “piko piko breath*” OR “soma breath*” OR “wim hof method” OR “oxygen advantage”)) |
|  | Holistic wellbeing AND breathing interventions | #6 | #4 AND #5 |
|  | Health conditions | #7 | (TI=(cancer OR patient* OR diabetes OR COPD OR asthma OR disease* OR cystic fibrosis OR myocardial infarction OR hypertension OR treatment)) |
|  | Holistic wellbeing AND breathing interventions NOT health conditions | #8 | #6 NOT #7 |
| Cochrane Library | Individual wellbeing | #1 | (wellbeing OR “well being” OR flourish* OR self NEXT connect* OR connect* NEXT to NEXT self OR connect* NEXT with NEXT self OR “self awareness” OR self NEXT accept* OR “self alignment” OR align* NEXT with NEXT *self OR “self determination” OR “self efficacy” OR “self esteem” OR “self identity” OR “self concept” OR consciousness OR interoception OR internal NEXT sensation* OR internal NEXT state* OR intuition* OR “true self” OR psychological* NEXT health OR “emotional health” OR “mental health” OR “life satisfaction” OR satisf* NEXT with NEXT life OR “positive affect” OR “negative affect” OR “physical health” OR positive NEXT emotion* OR negative NEXT emotion* OR joy OR “heart rate variability” OR HRV OR hedoni* OR eudaimoni* OR eudaemon* OR eudemo* OR happiness OR engag* OR meaning OR accomplishment OR achievement OR purpose OR “environmental mastery” OR “personal growth” OR matter* OR value* OR thriv* OR vitality OR resilience OR “quality of life” OR QoL OR autonomy OR competence):ti,ab,kw |
|  |  | #2 | MeSH descriptor: [Psychological Well-Being] explode all trees |
|  |  | #3 | MeSH descriptor: [Quality of Life] explode all trees |
|  |  | #4 | MeSH descriptor: [Heart Rate] explode all trees |
|  |  | #5 | MeSH descriptor: [Mental Health] explode all trees |
|  |  | #6 | #1 OR #2 OR #3 OR #4 OR #5 |
|  | Collective wellbeing | #7 | (“social wellbeing” OR “social well being” OR “social capital” OR “social capacity” OR “social participation” OR “social functioning” OR social NEXT actuali* OR “social inclusion” OR social NEXT intel* OR social NEXT accept* OR social NEXT identi* OR collective* OR psychosocial OR psycho NEXT social OR collaboration OR affiliation* OR community OR cohesi* OR belong* OR benevolen* OR relat* OR social NEXT support* OR friendship* OR connect* NEXT to NEXT others OR prosocial):ti,ab,kw |
|  |  | #8 | MeSH descriptor: [Social Status] explode all trees |
|  |  | #9 | MeSH descriptor: [Social Capital] explode all trees |
|  |  | #10 | MeSH descriptor: [Social Cohesion] explode all trees |
|  |  | #11 | MeSH descriptor: [Social Inclusion] explode all trees |
|  |  | #12 | MeSH descriptor: [Social Identification] explode all trees |
|  |  | #13 | MeSH descriptor: [Emotional Intelligence] explode all trees |
|  |  | #14 | MeSH descriptor: [Social Interaction] explode all trees |
|  |  | #15 | MeSH descriptor: [Social Participation] explode all trees |
|  |  | #16 | MeSH descriptor: [Interpersonal Relations] explode all trees |
|  |  | #17 | MeSH descriptor: [Altruism] explode all trees |
|  |  | #18 | #7 OR #8 OR #9 OR #10 OR #11 OR #12 OR #13 OR #14 OR #15 OR #16 OR #17 |
|  | Planetary wellbeing | #19 | (“planetary wellbeing” OR “planetary well being” OR “environmental wellbeing” OR “environmental well being” OR environment* NEXT attitude* OR environment* NEXT concern* OR environment* NEXT belief* OR environment* NEXT behav* OR environment* NEXT friend* OR environment* NEXT protect* OR environment* NEXT preserv* OR environment* NEXT steward* OR environment* NEXT identity* OR environment* NEXT interdepend* OR “ecological wellbeing” OR “ecological well being” OR ecolog* NEXT attitude OR “eco attitude” OR ecolog* NEXT behav* OR eco NEXT behav* OR ecolog* NEXT concern OR “eco concern” OR ecolog* NEXT belief OR “eco belief” OR pro NEXT environment* OR nature NEXT preserv* OR nature NEXT connect* OR nature NEXT relat* OR nature NEXT protect* OR “nature in self” nature NEXT behav* OR protection NEXT behav* OR sustainab* OR biophil* OR bio NEXT phil* OR climate NEXT friend* OR “climate conservation” OR climate* NEXT attitude* OR climate NEXT behav* OR climate NEXT belief* OR climate* NEXT aware* OR conservation* NEXT behav*):ti,ab,kw |
|  | Holistic wellbeing | #20 | #6 OR #18 OR #19 |
|  | Breathing interventions | #21 | (breathing NEXT intervention* OR breathing NEXT practice* OR breath* NEXT training OR breathing NEXT technique* OR “respiration practice” OR “breathing pattern” OR breathwork OR breath* NEXT work OR control* NEXT respiration OR control* NEXT breath* OR diaphragm* NEXT breath* OR abdom* NEXT breath* OR thoracic NEXT breath* OR chest NEXT breath* OR clavicul* NEXT breath* OR rhythmic NEXT breath* OR “functional breathing” OR belly NEXT breath* OR deep NEXT breath* OR slow NEXT breath* OR fast NEXT breath* OR shallow NEXT breath* OR nostril NEXT breath* OR nose NEXT breath* OR mouth NEXT breath* OR pursed NEXT lip NEXT breath* OR 4 NEXT 7 NEXT 8 NEXT breath* OR 4 NEXT 4 NEXT 8 NEXT breath* OR 2 NEXT 4 NEXT breath* OR five NEXT finger NEXT breath* OR resonant NEXT breath* OR coherent NEXT breath* OR paced NEXT breath* OR pranayama OR yogic NEXT breath* OR yoga NEXT breath* OR “sudarshan kriya yoga” OR kapalabhati OR “kapal bhati” OR skull NEXT shining NEXT breath* OR nadi NEXT shodan* OR nadi NEXT shodhan* OR “nadi sodhana” OR alternate NEXT nostril NEXT breath* OR “alternate nostril technique” OR “Nadi sudi” OR “nadi suddhi” OR “nadi shuddhi” OR “anulom viloma” OR viloma OR anulomviloma OR nerve NEXT purify* NEXT breath* OR channel NEXT cleaning NEXT breath* OR ujjayi OR ocean NEXT breath* OR victorious NEXT breath* OR bhramari OR bee NEXT breath* OR humming NEXT breath* OR dirga OR dirgha OR three NEXT part NEXT breath* OR three NEXT part NEXT breath* OR circular NEXT breath* OR infant NEXT breath* OR bhastrika OR bellow* NEXT breath OR sitali OR sheetali OR sheethali OR cooling NEXT breath* OR sheetkari OR sitkari OR sipping NEXT breath* OR hissing NEXT breath* OR samavritti OR “sama vritti” OR square NEXT breath* OR equal NEXT breath* OR box NEXT breath* OR plavini OR floating NEXT breath* OR murcha NEXT breath* O moorcha NEXT breath* OR swooning NEXT breath* OR “surya bhedana” OR suryabhedana OR sun NEXT piercing NEXT breath* OR “surya nadi” OR “chandra bhedana” OR chandrabhedana OR moon NEXT piercing breath* OR “chandra nadi” OR “sukka purvaka” OR “shwasa prashwasa” OR gita NEXT prana* OR sukha NEXT prana* OR easy* NEXT breath* OR “nisshesha rechaka kumbhaka” OR shunyaka OR “bahya” OR complete NEXT exhal* OR “outer breath retention” OR “antara kumbhaka” OR “internal breath retention” OR “kevala kumbhaka” OR “absolute retention” OR “spontaneous retention” OR “complete retention” OR “breath of fire” OR “agni prana” OR fire NEXT breath* OR simha* NEXT breath OR lion* NEXT breath* OR qigong NEXT breath* OR wave NEXT breath* OR xingqi OR circulating NEXT breath* OR “kidney breathing” OR lower NEXT back NEXT breath* OR conscious* NEXT connected NEXT breath* OR quantum NEXT light NEXT breath* OR *tummo NEXT breath* OR pico NEXT pico NEXT breath* OR piko NEXT piko NEXT breath* OR soma NEXT breath* OR “wim hof method” OR “oxygen advantage”):ti,ab,kw |
|  |  | #22 | MeSH descriptor: [Breathing Exercises] explode all trees |
|  |  | #23 | #21 OR #22 |
|  | Holistic wellbeing AND breathing interventions | #24 | #20 AND #23 |
|  | Health conditions | #25 | (cancer OR patient* OR diabetes OR COPD OR asthma OR disease* OR cystic fibrosis OR myocardial infarction OR hypertension OR treatment):ti,ab,kw |
|  | Holistic wellbeing AND breathing interventions NOT health conditions | #26 | #24 NOT #25 |

| Grey literature | | | |
| --- | --- | --- | --- |
| ProQuest | Individual wellbeing | S1 | title(Wellbeing OR “well being” OR well-being OR flourish* OR self-connect* OR “connect* to self” OR “connect* with self” OR self-awareness OR self-accept* OR self-alignment OR “align* with self” OR self-determination OR self-efficacy OR self-esteem OR self-identity OR self-concept OR consciousness OR interoception OR “internal sensation*” OR “internal state*” OR intuition* OR “true self” OR “psychological* health” OR “emotional health” OR “mental health” OR “life satisfaction” OR “satisf* with life” OR “positive affect” OR “negative affect” OR “physical health” OR “positive emotion*” OR “negative emotion*” OR joy OR “heart rate variability” OR HRV OR hedoni* OR euda*moni* OR eudemon* OR happiness OR engag* OR meaning OR accomplishment OR achievement OR purpose OR “environmental mastery” OR “personal growth” OR matter* OR value* OR thriv* OR vitality OR resilience OR “quality of life” OR QoL OR autonomy OR competence) |
|  | Collective wellbeing | S2 | title(“social wellbeing” OR “social well-being” OR “social well being” OR “social capital” OR “social capacity” OR “social participation” OR “social functioning” OR “social actual*” OR “social inclusion” OR “social intel*” OR “social accept*” OR “social identi*” OR “social connect*” OR collective* OR psychosocial OR psycho-social OR collaboration OR affiliation* OR community OR cohesi* OR belong* OR benevolen* OR relat* OR “social support*” OR friendship* OR “connect* to others” OR prosocial) |
|  | Planetary wellbeing | S3 | title(“planetary wellbeing” OR “planetary well being” OR “environmental wellbeing” OR “environmental well being” OR “environmental attitude” OR “environmental concern” OR “environmental belief” OR “environmental behaviour” OR “environmental friendship” OR “environmental protection” OR “environmental preservation” OR “environmental stewardship” OR “environmental identity” OR “environmental interdependence” OR “ecological wellbeing” OR “ecological well being” OR “ecological attitude” OR “eco attitude” OR “ecological behaviour” OR “eco behaviour” OR “ecological concern” OR “eco concern” OR “ecological belief” OR “eco belief” OR pro-environment OR “pro environment” OR “nature preservation” OR “nature connection” OR “nature relatedness” OR “nature protect” OR “nature in self” OR “nature behaviour” OR “preservation behaviour” OR “protection behaviour” OR sustainability OR sustainable OR biophilia OR “bio philia” OR “climate friendship” OR “climate conservation” OR “climate change attitude” OR “climate change behaviour” OR “climate change belief” OR “climate change awareness” OR “conservation behaviour”) |
|  | Holistic wellbeing | S4 | [S1] OR [S2] OR [S3] |
|  | Breathing interventions | S5 | title(“breathing intervention*” OR “breathing practice*” OR “breath* training” OR “breathing technique*” OR “breathing exercise*” OR “respiration practice” OR “breathing pattern” OR breathwork OR “breath* work” OR “control* respiration” OR “control* breath*” OR “diaphragm* breath*” OR “abdom* breath*” OR “thoracic breath*” OR “chest breath*” OR “clavicul* breath*” OR “rhythmic breath*” OR “functional breath*” OR “belly breath*” OR “deep breath*” OR “slow breath*” OR “fast breath*” OR “shallow breath*” OR “nostril breath*” OR “nose breath*” OR “mouth breath*” OR “pursed lip breath*” OR “pursed-lip breath*” OR “4 7 8 breath*” OR “4-7-8 breath*” OR “4 4 8 breath*” OR “4-4-8 breath*” OR “2-4 breath*” OR “five-finger breath*” OR “resonant breath*” OR “coherent breath*” OR “paced breath*” OR pranayama OR “yogic breath*” OR “yoga breath*” OR “sudarshan kriya yoga” OR kapalabhati OR “kapal bhati” OR “skull shining breath*” OR “nadi shodan*” OR “nadi shodhan*” OR “nadi sodhana” OR “alternate nostril breath*” OR “alternate nostril technique” OR “nadi sudi” OR “nadi suddhi” OR “nadi shuddhi” OR “anulom viloma” OR viloma OR “anulomviloma” OR “nerve purify* breath*” OR “channel cleaning breath*” OR ujjayi OR “ocean breath*” OR “victorious breath*” OR bhramari OR “bee breath*” OR “humming breath*” OR dirga OR dirgha OR “three part breath*” OR “three-part breath*” OR “circular breath*” OR “infant breath*” OR bhastrika OR “bellow* breath*” OR sitali OR sithali OR sheetali OR “cooling breath*” OR sheetkari OR sitkari OR “sipping breath*” OR “hissing breath*” OR samavritti OR “sama vritti” OR “square breath*” OR “equal breath*” OR “box breath*” OR plavini OR “floating breath*” OR “murcha breath*” OR “moorcha breath*” OR “swooning breath*” OR “surya bhedana” OR suryabhedana OR “sun piercing breath*” OR “surya nadi” OR “chandra bhedana” OR chandrabhedana OR “moon piercing breath*” OR “chandra nadi” OR “sukka purvaka” OR “shwasa prashwasa” OR “gita prana*” OR “sukha prana*” OR “easy* breath*” OR “nisshesha rechaka kumbhaka” OR shunyaka OR “bahya” OR “complete exhal*” OR “outer breath retention” OR “antara kumbhaka” OR “internal breath retention” OR “kevala kumbhaka” OR “absolute retention” OR “spontaneous retention” OR “complete retention” OR “breath of fire” OR “agni prana” OR “fire breath*” OR “simha* breath*” OR “lion* breath*” OR “qigong breath*” OR “wave breath*” OR “xingqi” OR “circulating breath*” OR “kidney breathing” OR “lower back breath*” OR “conscious* connected breath*” OR “quantum light breath*” OR “tummo” OR “pico pico breath*” OR “piko piko breath*” OR “soma breath*” OR “wim hof method” OR “oxygen advantage”) |
|  | Holistic wellbeing AND breathing interventions | S6 | [S4] AND [S5] |
|  | Health conditions | S7 | title(cancer OR patient* OR diabetes OR COPD OR asthma OR disease* OR cystic fibrosis OR myocardial infarction OR hypertension OR treatment) |
|  | Holistic wellbeing AND breathing interventions NOT health conditions | S8 | [S6] NOT [S7] |
| Google Scholar | Individual |  | (“breathing intervention” OR “breathing technique” OR “breathing practice” OR “breathing exercise” OR breathing OR pranayama OR breathwork OR “breath control” OR pranayam OR tummo) AND (wellbeing OR flourishing OR thriv OR “heart rate” OR self OR interoception OR “mental health” OR emotion OR “psychological health” OR “life satisfaction” OR autonomy OR affect OR “physical health” OR hedon OR eudaim OR happiness OR engagement OR meaning OR accomplishment OR purpose OR achievement OR “environmental mastery” OR “personal growth” OR mattering OR joy OR value OR vitality OR optimism OR “quality of life” OR competence OR resilience) -patient |
|  | Collective |  | (“breathing intervention” OR “breathing technique” OR “breathing practice” OR “breathing exercise” OR breathing OR pranayama OR breathwork OR “breath control” OR pranayam OR tummo) AND (“collective wellbeing” OR social OR psychosocial OR collaboration OR affiliation OR community OR cohesion OR belonging OR relationships OR friendship OR “connection to others” OR benevolence OR prosocial) -patient |
|  | Planetary |  | (“breathing intervention” OR “breathing technique” OR “breathing practice” OR “breathing exercise” OR breathing OR pranayama OR breathwork OR “breath control” OR pranayam OR tummo) AND (“planetary wellbeing” OR environment OR ecological OR conservation OR climate OR biophilia OR sustainability OR sustainable OR nature OR biophilia) -patient |
| PsyArXiv | All |  | “breathing” AND wellbeing |
