# Supplementary material for: Breathwork and holistic wellbeing: A protocol for a scoping review
Source: PLoS One. 2025 Sep 30;20(9):e0333360. doi: 10.1371/journal.pone.0333360 (PMC12483246; doi:10.1371/journal.pone.0333360)
Supplement: S3 Table — (DOCX) [file pone.0333360.s003.docx]

**S3 Table. Study concepts and associated search terms**

| **Concept** | **Category** | **Variations** |
| --- | --- | --- |
| Breathing interventions | Generic references to breathing interventions | 2-4 breathing  4-4-8 breathing  4-7-8 breathing  Abdominal breathing  Belly breathing  Breath control  Breathing exercises  Breathing intervention  Breathing pattern  Breathing practice  Breathing training Breathing technique  Breathwork  Chest breathing  Clavicular breathing  Coherent breathing  Deep breathing  Diaphragmatic breathing  Fast breathing  Five finger breathing  Functional breathing  Mouth breath/ing  Nose breath/ing  Paced breathing  Pursed lip breath/ing  Resonant breathing  Respiration practice  Rhythmic breathing  Shallow breathing  Slow breathing  Thoracic breathing |
|  | Prāṇāyāma (Yoga) | Agni Prana/Breath of fire/Fire breathing  Antara kumbhaka/Internal breath retention  Bhastrika/Bellows breath  Bhramari/Humming bee breath  Chandra bhedana/Chandra bheda/Moon piercing breath/Left nostril breathing/Chandra nadi  Dirga/Three-part breath/Circular breathing/Infant breathing  Kapalabhati/Skull shining breath  Kevala kumbhaka/Absolute retention/Spotaneous retention/Complete retention  Murcha/Swooning breath  Nadi s(h)odana/Nadi suddhi/Nadi Shuddhi/Alternate nostril breathing/Anulom vilom/Nerve purifying breath/Channel cleaning breath  Nisshesha rechaka kumbhaka/Shunyaka/Bahya kumbhaka/Complete exhalation/Outer breath retention  Plavini/Floating breath  Prāṇāyāma  Samavritti/Square breathing/Equal movement breathing/Box breathing  Sheetali/Sitali/Sithali/Cooling breath  Sheetkari/Sitkari/Sipping breath/Hissing breath  Simhasana/Simha/Lion’s breath  Sudarshan kriya yoga (SKY)  Sukka purvaka/Shwasa prashwasa/Gita/Sukha/Easy comfortable breathing  Surya bhedana/Sun piercing breath/Right nostril breathing/Surya nadi  Ujjayi/Ocean breathing/Victorious breath  Yogic breath/ing |
|  | Chinese Qigong | Diaphragmatic breathing/Kidney breathing/Lower back breathing/Deep abdominal breathing/Belly breathing  Qigong breathing  Wave breathing/Three-part breath  Xingqi/Circulating breathing |
|  | Conscious Connected Breathing | Biodynamic breathwork  Breathwork transformation  Conscious connected breathing  Holotropic/Grof® breathwork  Quantum Light Breath  Rebirthing breathwork  Transformational breathwork |
|  | g-tummo (Tibetan Buddhism) | g-tummo |
|  | Piko piko breath (Hawaiian Kahuna) | Pico pico breath |
|  | Others | SOMA Breath®  The Oxygen Advantage ®  Wim Hof Method (prāṇāyāma only) |
| **Holistic wellbeing** | Individual wellbeing | Accomplishment  Achievement  Autonomy  Competence  Consciousness  Emotional health  Emotional wellbeing  Engagement  Environmental mastery  Eudaimonia  Flourishing  Happiness  Heart rate variability (HRV)  Hedonia  Interoception  Joy  Life satisfaction  Mattering  Meaning  Mental health  Negative affect (reduce)  Negative emotion(s) (reduce)  Personal growth  Physical health  Positive affect  Positive emotion(s)  Psychological health  Psychological wellbeing  Purpose  Quality of life  Resilience  Self-acceptance  Self-alignment  Self-awareness  Self-concept  Self-connection  Self-determination  Self-efficacy  Self-esteem  Self-identity  Subjective wellbeing  Thriving  True self  Value(s)  Vitality  Wellbeing |
|  | Collective wellbeing | Affiliation  Belonging  Benevolence  Collaboration  Collective wellbeing  Community involvement  Community participation  Cohesion  Connection to others  Friendship  Prosocial behaviour  Psychosocial factors  Relationships  Social acceptance  Social actualisation  Social capacity  Social capital  Social connection  Social functioning  Social identity  Social inclusion  Social intelligence  Social participation  Social support  Social wellbeing |
|  | Planetary wellbeing | Biophilia  Climate change attitude(s)  Climate change awareness  Climate change behaviour(s)  Climate change belief(s)  Climate conservation  Climate friendliness  Conservation behaviour(s)  Ecological attitude(s)  Ecological behaviour(s)  Ecological belief(s)  Ecological concern(s)  Ecological wellbeing  Environmental attitude(s)  Environmental behaviour(s)  Environmental concern(s) Environmental belief(s)  Environmental friendliness  Environmental identity  Environmental interdependence  Environmental preservation  Environmental protection  Environmental stewardship  Environmental wellbeing  Nature behaviour  Nature connectedness  Nature in self  Nature preservation  Nature protection  Nature relatedness  Nature relationship  Planetary wellbeing  Preservation behaviour(s)  Pro-environmental attitude(s)  Pro-environmental behaviour(s)  Protection behaviour(s)  Sustainability  Sustainable behaviour(s) |
