# Supplementary material for: Breathwork and holistic wellbeing: A protocol for a scoping review
Source: PLoS One. 2025 Sep 30;20(9):e0333360. doi: 10.1371/journal.pone.0333360 (PMC12483246; doi:10.1371/journal.pone.0333360)
Supplement: S5 Table — (DOCX) [file pone.0333360.s005.docx]

**S5 Table. Non-exhaustive selection of potential breathing interventions**

| **Breathing technique** | **Founder/teacher, researcher or tradition** | **Brief description** | **Technique speed** |
| --- | --- | --- | --- |
| Kapalabhati (skull shining breath) | Yoga (prāṇāyāma) [1] | Steady repetition of forceful exhalations through the nose while contracting the stomach muscles, with passive inhales [1]. | Fast |
| Nadi Shodhana (alternate nostril breathing) | Yoga (prāṇāyāma) [1] | Alternating breathing from one nostril at a time in a specific pattern [1]. | Slow |
| Bhramari (humming bee breath) | Yoga (prāṇāyāma) [1] | Inhaling deeply through the nose and humming with lips closed while exhaling [1]. | Slow |
| Dirga (three-part breath) | Yoga (prāṇāyāma) [1] | Breathing into the abdomen, chest and clavicular region [1]. | Slow |
| Ujjayi (ocean’s breath) | Yoga (prāṇāyāma) [1] | Inhaling and exhaling with a slight constriction at the throat [1]. | Slow |
| Samavritti (equal movement / box breathing) | Yoga (prāṇāyāma) [1] | Equal lengths inhalation, exhalations and retentions e.g. exhale for the count of four, retention for four, inhale for four, retention for four [1]. | Slow |
| Holotropic / Grof® breathwork | Stanislav Grof and Cristina Grof [2] | Uses conscious connected breathing style; rapid, deep breathing with evocative music, focus on a forceful exhale [2]. | Fast |
| Transformational Breathwork | Dr. Judith Kravitz [3] | Active deep abdominal breathing through the mouth with music [3]. | Fast |
| Rebirthing Breathwork / Conscious Connected Breathing | Leonard Orr [4] | Uses conscious connected breathing style; fast and deep breathing, focusing on deep inhale, typically increasing in speed, without pause between inhale and exhale [4]. | Both |
| g-tummo (inner fire breathing) | Tibetan Buddhism [5] | Deep inhalation, retain breath, and then contract abdominal and pelvic muscles to create a vase-like shame in lower belly, with visualisation of inner heat. Two main types: ‘Forceful Breath’ and ‘Gentle Breath’ with differing breathing techniques, visualisation and goal [5]. | Both |
| Resonance Breathing | Evgeny Vaschillo, Dr. Paul M. Lehrer [6] | Slow breathing to an individual’s ‘resonance frequency’, syncing with the baroreflex, around 4 to 7 breaths per minute [6]. | Slow |
| Coherent Breathing | Dr. Stephen Elliott [7] | Maintaining consistent breath rate, around 5 to 6 breaths cycles per minute, without pause [7]. | Slow |

**References**

1. Iyengar BKS. Light on Pranayama. Pranayama Dipika. London: Unwin Paperbacks; 1983.

2. Grof S, Grof C. Holotropic Breathwork: A New Approach to Self-Exploration and Therapy. Excelsior Editions; 2010.

3. Kravitz J. Breathe Deep, Laugh Loudly: The Joy of Transformational Breathing. Free Press Ink; 1999.

4. Orr L, Ray S. Rebirthing in the New Age. Celestial Arts; 1977.

5. Mullin G. Readings on Six Yogas of Naropa. Ithaca, NY: Snow Lion Publications; 1997.

6. Lehrer PM, Vaschillo E, Vaschillo B. Resonant frequency biofeedback training to increase cardiac variability: rationale and manual for training. Appl Psychophysiol Biofeedback. 2000;25: 177–191. doi:10.1023/a:1009554825745

7. Elliott S, Edmonson D. Coherent Breathing: The Definitive Method - Theory and Practice. Coherence Press; 2008.
